# Reduction of Interhemispheric Functional Brain Connectivity in Early Blindness: A Resting-State fMRI Study

**DOI:** 10.1155/2017/6756927

**Published:** 2017-06-01

**Authors:** Fen Hou, Xia Liu, Zhifeng Zhou, Jingyi Zhou, Hengguo Li

**Affiliations:** Medical Imaging Center, The First Affiliated Hospital of Jinan University, Guangzhou 510632, China

## Abstract

**Objective:**

The purpose of this study was to investigate the resting-state interhemispheric functional connectivity in early blindness by using voxel-mirrored homotopic connectivity (VMHC).

**Materials and Methods:**

Sixteen early blind patients (EB group) and sixteen age- and gender-matched sighted control volunteers (SC group) were recruited in this study. We used VMHC to identify brain areas with significant differences in functional connectivity between different groups and used voxel-based morphometry (VBM) to calculate the individual gray matter volume (GMV).

**Results:**

VMHC analysis showed a significantly lower connectivity in primary visual cortex, visual association cortex, and somatosensory association cortex in EB group compared to sighted controls. Additionally, VBM analysis revealed that GMV was reduced in the left lateral calcarine cortices in EB group compared to sighted controls, while it was increased in the left lateral middle occipital gyri. Statistical analysis showed the duration of blindness negatively correlated with VMHC in the bilateral middle frontal gyri, middle temporal gyri, and inferior temporal gyri.

**Conclusions:**

Our findings help elucidate the pathophysiological mechanisms of EB. The interhemispheric functional connectivity was impaired in EB patients. Additionally, the middle frontal gyri, middle temporal gyri, and inferior temporal gyri may be potential target regions for rehabilitation.

## 1. Introduction

Blind patients usually rely on nonvisual information in external environments for cognition, perception, and mobility, especially when their visual functions were deprived at an early age [1]. Indeed, patients often place higher regard to the nonvisual signals and process them more effectively than sighted individuals. This adaption may be a result of structural and functional remodeling or reorganization of the brain functional cortices. Previous studies noted that early visual deprivation has the ability to remodel the visual cortex for cross-modal signal processing, helping patient perception with nonvisual approaches including tactile [2], auditory [3, 4], and olfactory sensation [5]. Additionally, some studies have observed that the baseline metabolism and blood flow in the visual cortex are increased in early blind patients [6]. A previous study demonstrated that early blindness showed resting-state correlations between V1 and V2/V3 in retinotopic pattern [7]. There are also propositions that early blind patients exhibit stronger auditory and parietal networks and weaker vision-related occipital networks compared with sighted subjects [8]. Meanwhile, the congenital blindness (CB) and the late blindness (LB) showed decreased short- and long-range functional connectivity densities in the primary visual cortex (V1) [9].

Diffusion tensor imaging (DTI) has been widely used to investigate the microstructural properties of white matters in visual neuroscience [10]. The topological properties of the anatomical brain network such as connectivity density and global efficiency is decreased [11], and that resting-state brain functional connectivity is also altered [12]. Previous diffusion tensor imaging (DTI) investigations have confirmed white-matter reorganization in the splenium of the corpus callosum in patients with early blindness [13, 14]. The white-matter tracts in the corpus callosum connect the bilateral hemispheres of the brain and facilitate interhemispheric integration and communication [15]. The white-matter reorganization in the splenium of the corpus callosum probably disrupts the interhemispheric functional and structural connectivity [16]. Communication and coordination between the bilateral hemispheres are crucial for information processing in the brain. Based on these previous findings, investigating the definitive alteration of interhemispheric functional connectivity is important in determining the pathophysiologic mechanism of early blindness.

Voxel-mirrored homotopic connectivity (VMHC) is a new methodology for analyzing interhemispheric functional connectivity on the basis of resting-state functional magnetic resonance imaging (rs-fMRI) [17]. VMHC allows quantification of the interhemispheric homotopic connections by calculating brain functional connectivity between a voxel and its mirrored counterpart located in the bilateral hemispheres. In previous studies, this modality has been widely applied in a variety of psychiatric and neurological conditions including schizophrenia [18], cocaine addiction [19], and tinnitus [20]. To our knowledge, this is the first study that VMHC has been applied to evaluate early blindness.

Herein, we assessed interhemispheric functional coordination in early blind patients and further analyze the correlation between altered interhemispheric functional connectivity and clinical features of early blindness.

## 2. Materials and Methods

### 2.1. Subjects

This study was approved by the Medical Research Ethics Committee of Jinan University, China. The early blindness was defined as blindness with onset < 2 years of age [21]. Sixteen early blind patients were enrolled from the Guangdong Province Blind School (age ranging from 11 years to 18 years; duration of blindness ranging from 132 months to 213 months). The demographic data of the early blind patients are summarized in Table 1. Sixteen age- and gender-matched sighted individuals with corrected visual acuity higher than 0.8 were recruited as normal controls. The inclusion criteria included (1) right-handedness and (2) age < 19 years. The exclusion criteria included (1) any history of psychiatric or neurologic diseases and (2) identifiable MRI abnormalities, such as vascular malformations or tumors. All individuals signed written informed consent form.

### 2.2. Data Acquisition

The functional and structural magnetic resonance images in patients and sighted controls were acquired using a 3.0-Tesla magnetic resonance scanner (GE Discovery 750, USA). To minimize head motion and to reduce scanner noise, we used fitting foam padding and earplugs. T1-weighted anatomic images were collected using a 3D BRAVO sequence; the parameters included the following: TR = 8.2 ms; TE = 3.2 ms; FA = 12°; matrix size = 256 × 256; FOV = 256 × 256 mm^2^; slice thickness = 1 mm; and 172 slices in the axial plane. An 8-minute rs-fMRI scanning was conducted using a gradient echo-planar sequence; the parameters were set as follows: 240 time points; TR = 2000 ms; TE = 35 ms; FA = 90°; matrix size = 64 × 64; FOV = 256 × 256 mm^2^; slice thickness = 3 mm; gap = 0.6 mm; and 41 slices in the axial plane. During fMRI scans, all subjects were instructed to keep their eyes closed, relax, do not move, think of nothing in particular, and stay awake. After the fMRI scan, the fMRI images and subjects' conditions should be checked to make sure whether they satisfied the requirements; if not, the fMRI data were abandoned and scanned again.

### 2.3. Data Preprocessing

VMHC analysis: we preprocessed rs-fMRI data using the Data Processing Assistant for rs-fMRI (DPARSF, http://resting-fmri.sourceforge.net) and Statistical Parametric Mapping software package (SPM8, http://www.fil.ion.ucl.ac.uk/spm). We discarded the first ten volumes to eliminate the nonequilibrium effects of magnetization and to allow the participants' environment adaptation. In the remaining images, we corrected the time delay and head motion. The motion threshold was set as <2.0 mm spatial shift or <2.0° rotation in any direction. After motion correction, the anatomic images of each subject were registered to the realigned echo-planar images. A T1-weighted imaging template (http://www.bic.mni.mcgill.ca/ServicesAtlases) was used for adolescent subjects [22]. Subsequently, we used a 4 mm full width half maximum Gaussian kernel for spatial smoothing. We then performed a temporally bandpass filter at 0.01–0.08 Hz and linear trend removal. Furthermore, we used linear regression to remove confounding factors, including six head motion parameters and ventricle and white matter signals.

Voxel-based morphometry (VBM) analysis: in order to avoid the effect of structural differences on VMHC measurements, morphometric analysis was performed using a VBM approach to calculate individual gray matter volume (GMV). Data preprocessing was conducted with the SPM8 software package running on a Matlab 12 platform (MathWorks, Natick, MA, USA). The white matter, gray matter, and cerebrospinal fluid were identified on images according to the standard unified segmentation module. Population templates for gray matter were derived from the whole imaging data set according to the protocol described in the literature [23]. Subsequently, imaging data were normalized to the MNI space according to the pediatric T1-weighted template. Next, we used a 4 mm full width half maximum Gaussian kernel for spatial smoothing.

### 2.4. Statistical Analysis and Imaging Data Calculation

All demographics analyses were performed using Statistical Package for the Social Science (SPSS) software (version 19.0; IBM Corporation, NY, USA). Chi-squared test was used for gender comparison, and two-sample *t*-test was used for comparing intergroup demographic differences. A two-tailed *P* value less than 0.05 was considered statistically significant.

#### 2.4.1. VMHC Data Analysis

VMHC was computed using the REST software (http://resting-fmri.sourceforge.net). We calculated VMHC according to the protocols described previously by Zuo et al. [17]. Prior to group analysis, we used a Gaussian kernel for spatial smoothing. We entered the transformed *Z*-maps of each subject into a random-effect two-sample *t*-test model to identify the VMHC difference between EB group and sighted control group, setting the age and gender as covariates. The statistical threshold was set at AlphaSim corrected *P* value < 0.05 and cluster size > 85. For further group analysis, we controlled the bilateral differences in GMV by setting the 3D VMHC volume as a covariate [20].

#### 2.4.2. VBM Calculation

The preprocessed images of each subject were collected and analyzed using the SPM8 statistical tool. A random-effect two-sample *t*-test model was used, with the age and gender as covariates. The statistical threshold was set at AlphaSim corrected *P* value < 0.05 and cluster size > 85.

#### 2.4.3. Correlation Analysis

Correlation analyses were conducted using REST software. Pearson's test was used to analyze the correlation between the VMHC and duration of blindness. The age, gender, and GMV were set as nuisance covariates. The statistical threshold was set at AlphaSim corrected *P* value < 0.01 and cluster size > 18.

## 3. Results

### 3.1. Demographic Characteristics

This study enrolled 11 male and 5 female patients with early blindness, with an average age of 14.8 ± 2.1 years. The recruited sighted control subjects were age and gender matched. Statistically, no significant differences in the demographic characteristics were noted between the early blind (EB) group and sighted control (SC) group (*P* > 0.05). The detailed data are summarized in Table 2.

### 3.2. Visual Cortex VMHC Analysis

**Figure 1:**
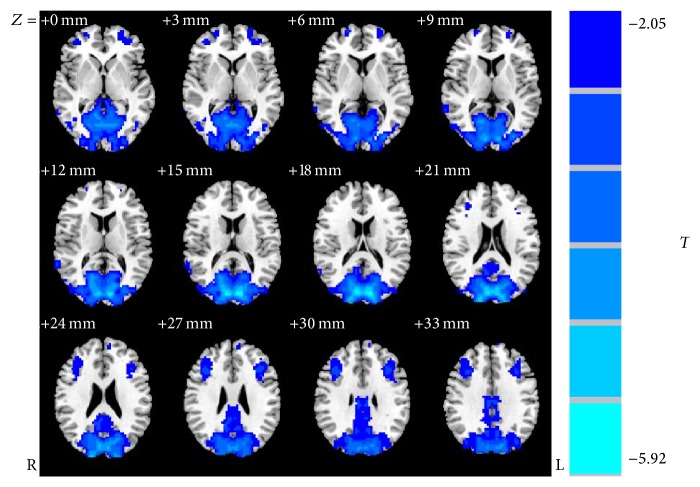
Regions showing significant differences in voxel-mirrored homotopic connectivity between groups: blue regions denoting areas with lower voxel-mirrored homotopic connectivity in early blind group when compared to sighted controls.

When compared to the SC group, the EB group had significantly lower VMHC in primary visual cortex, visual association cortex, and somatosensory association cortex (*P* < 0.05). The involved brain areas included the bilateral calcarine cortices, lingual cortices, cuneus, superior occipital gyri, middle occipital gyri, and precentral cortices. The results are presented in Table 3 and Figure 1. Additionally, no brain area with increased VMHC was detected in EB group.

### 3.3. Visual Cortex VBM Analysis

**Figure 2:**
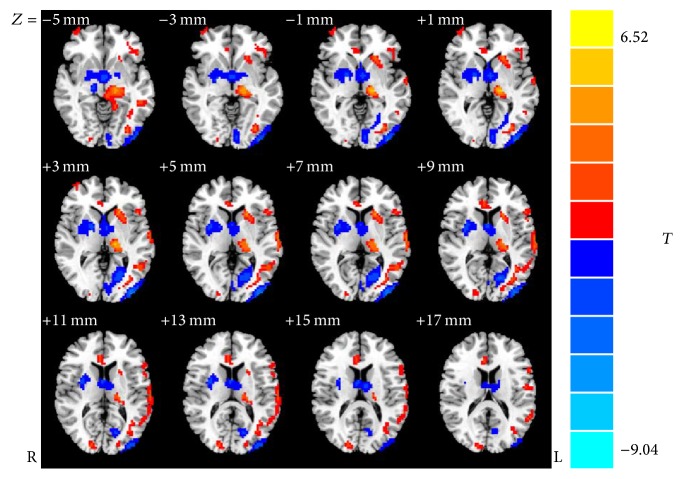
Voxel-based morphometry analysis of group effects on regional brain gray matter, blue regions denoting areas with lower GMV and red regions denoting areas with greater GMV in early blind group when compared to sighted controls. The numbers at the bottom of the figure indicating the Montreal Neurological Institute coordinates of the slices.

VBM analysis showed the GMV of the left lateral calcarine cortices in EB group was significantly smaller than that in the SC group (*P* < 0.05); nevertheless, the GMV of the left lateral middle occipital gyri was significantly larger (*P* < 0.05). The detailed results are present in Table 4 and Figure 2.

### 3.4. Correlation Analysis

**Figure 3:**
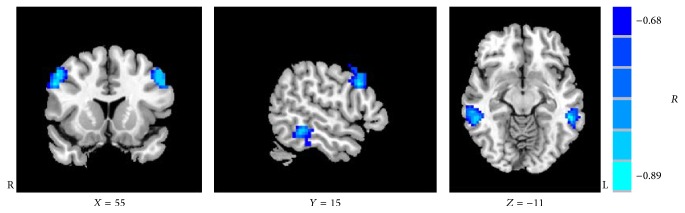
Regions showing significant correlations between voxel-mirrored homotopic connectivity and duration of blindness.

Pearson correlation analyzes the durations of blindness negatively correlated with VMHC in the bilateral middle frontal gyri, middle temporal gyri, and inferior temporal gyri (*P* < 0.01) (Table 5 and Figure 3). After correction for gender, age, and GMV, Pearson correlation analyses showed the negative correlation as well (*P* < 0.01).

## 4. Discussions

Over the past twenty years, rs-fMRI has been shown to be an effective tool to evaluate the functional connectivity of the human brain and has been widely applied in many areas, such as various neurological and psychiatric abnormalities. Homotopic functional connectivity is an important parameter that reflects cerebral intrinsic functional architecture. Interhemispheric connectivity has revealed the exchanging and the processing of information between the bilateral brain hemispheres. VMHC is a new proposed approach to assess the homotopic functional connectivity, which makes it feasible to calculate resting-state brain functional connectivity between each voxel and its mirrored counterpart located in the bilateral hemisphere. Indeed, changes in interhemispheric functional connectivity have been noted both in clinical psychiatric disorders and in healthy aged individuals [17]. However, electrophysiological and neuroimaging studies focusing on early blindness are, to date, sparse. To the best of our knowledge, this is the first study that VMHC has been applied to identify the alterations of homotopic functional connectivity in adolescent early blind patients. Herein, we investigated the interhemispheric functional interactions and analyzed the relevant correlation with clinical characteristics of early blindness.

Our findings revealed significantly decreased VMHC in the bilateral calcarine cortices, lingual cortices, cuneus, superior occipital gyri, middle occipital gyri, and precentral cortices in EB group. Furthermore, VMHC in the bilateral middle frontal gyri, middle temporal gyri, and inferior temporal gyri negatively correlated with the duration of blindness. Moreover, a further VBM analysis revealed a significantly reduced GMV in the left lateral calcarine cortices and a significantly increased GMV in the left lateral middle occipital gyri. These results indicated that interhemispheric connectivity was significantly reduced in the whole visual system of EB patients when compared to the SC control group. Our findings suggested that the whole occipital lobe was involved, including BA17 and BA18 areas and superior extrastriate areas. VMHC reductions in the visual functional regions were extensive, especially in the striate cortex (BA17 area), in the extrastriate cortex (BA18/19 areas), and in the precentral cortex (BA7 area), which suggested that the interhemispheric synchronization of spontaneous neural activity was decreased in these brain areas, and the functional homology of the primary visual cortex, visual association, and somatosensory association might be impaired in adolescent early blind patients. According to previously published findings, reports have proposed that reduced interhemispheric communication could be interpreted as dysfunctional connectivity, and speculated various pathophysiological mechanisms involving a specific brain area, such as disturbed synaptic modulation, might be the triggers [24]. Thus, heterogeneity in oscillations of neuronal elements could result in a reduction of interhemispheric connectivity [24]. Other research has indicated that the deficits in VMHC might be related to extensive abnormalities in white-matter integrity, in particular, those in the corpus callosum [25, 26]. The corpus callosum is a crucial conduction system for interhemispheric information interactions and coordination; and the damage of the corpus callosum greatly impairs the interhemispheric transfer of information, leading to attentional and cognitive disturbances. Indeed, a study by Shimony et al. examined congenitally blind patients using DTI techniques and observed white-matter reorganization manifesting as atrophy of the afferent projections to the visual cortex including the inferior longitudinal tract, the geniculostriate system, and the posterior part of the corpus callosum [13]. According to current findings, the information integration between the bilateral visual fields may be mediated by the splenium, the white matter of the posterior corpus callosum [10]. Thus, we hypothesized that the white-matter reorganization in the splenium of the corpus callosum might be responsible for the reduction of interhemispheric connectivity between bilateral visual cortices in adolescent early blind patients.

VBM is an advanced neuroimaging calculation method using statistical parametric mapping, which provides measurements of focal differences in brain anatomy [27]. Traditional morphometry, which measures volume of the whole brain or specific areas using regions of interest on imaging maps and calculating the enclosed volume, only allows measurements of large brain areas but overlooks small differences in focal volume. This modality makes it possible to characterize differences on a voxel-by-voxel basis. VBM not only is used for examining differences across subjects but is also widely used to examine neuroanatomical differences between hemispheres detecting structural homology and brain asymmetry. Recently, VBM has been widely applied to evaluate structural differences in brain anatomy in various disorders, such as Alzheimer's disease and Parkinsonism [28, 29]. Furthermore, VBM studies have also demonstrated brain structure changes in blind individuals. Earlier morphometric studies conducted by Jiang et al. on early blind patients and observed gray matter atrophy in the left lateral calcarine cortices [1], which is similar to our findings. The reduction of GMV in the BA17 primary visual cortex in early blindness has been construed as an adaptive response to loss of peripheral visual input [30]. Additionally, consistent with our findings, Noppeney also noted the involvement of extrastriate cortices, which is responsible for higher-level semantic retrieval processing, in early blind patients [31]. The reduction of GMV in primary visual cortex and the increase of GMV in left lateral middle occipital gyrus in early blindness, when compared to the SC group, may be due to both degeneration and cross-modal functional plasticity. When the visual input was absent, the primary visual cortex was significantly more degenerated than the extrastriate cortices, but its potential for cross-modal plasticity is lower than that of extrastriate cortices [31, 32]. This phenomenon may be explained by the fact that the primary visual cortices developed later and depend on visual experience, receiving visual information. In contrast, extrastriate cortices developed earlier and are not sensitive to visual dependence, receiving a larger input from others polymodal sensory (nonvisual) information. In the current study, more than half of the early blind subjects who never had any visual experiences could not receive visual information, so the GMV in the left lateral calcarine cortices (primary visual cortex) showed significant atrophy. In contrast, cross-modal plasticity is more significant than degeneration in the left lateral middle occipital gyrus (extrastriate cortices), leading to an increased GMV. The definitive mechanism of the involvement of extrastriate cortices in early visual deprivation requires further investigation.

In the current study, we found the duration of blindness negatively correlated with VMHC in the bilateral middle frontal gyri, middle temporal gyri, and inferior temporal gyri, which indicated that interhemispheric interactions of these regions might be influenced by disease courses. Previous studies have described the reduction of white-matter connectivity between the occipital and temporal lobes in blindness; meanwhile the occipital cortex shows increased responses to language tasks. Recently, there has been an alternative perspective suggesting that the occipital cortex may compete rather than collaborate with nondeprived regions of cortex for different functions. For example, the lateral occipital cortex may compete with temporal areas with language tasks, leading to maximize the decoupling between two regions. It seemed that it was easy to understand that there were reductions in anatomical connectivity between the occipital cortex and temporal lobes. Therefore, the input from the occipital in the temporal lobes decreased compared with the sighted individuals and may lead to reduced connectivity between bilateral temporal lobes [10]. We speculate the reduction may progress with the duration of blindness, which may explain the correlation between VMHC and duration of the blindness. In the present study, early blind patients either lack visual experience with no residual light perception or have just a short-term visual experience, and the visual work memory continues to decline and the fiber connectivity of bilateral hemisphere reduces with the duration of lack of visual experience. Furthermore, visual cortical reorganization continued to increase, and the compensatory connectivity between bilateral visual cortices did not significantly reduce. On the other hand, some studies suggested that the interactions between the following brain regions may be involved in visual working memory: prefrontal cortex, medial temporal lobe, and inferior temporal lobe [33]. Medial temporal lobe and prefrontal cortex have been found to play a crucial role for maintaining item characters or manifold items in visual working memory via interactions with content-specific regions in inferior temporal lobe [34]. Thus, the bilateral middle frontal gyri, middle temporal gyri, and inferior temporal gyri may be potential target regions for rehabilitation of category-specific visual working memory in early blind patients.

The major limitation of the current study is the limited sample size, and further studies with larger cohorts are needed. Additionally, the human brain is asymmetrical in nature, while the current study used a symmetric standard template and the functional data were smoothed; future studies should consider data-driven approaches, such as clustering interhemispheric voxels on the basis of their resting-sate functional connectivity, to define functional homotopy. The findings in the present study shed light on the role that structural changes may play in VMHC alterations in early blindness. In future studies, DTI should be considered as a promising approach to detect changes in anisotropy within specific fiber tracts and the relevant correlation with VMHC.

## 5. Conclusions

This is the first study using VMHC to identify the alterations of homotopic functional connectivity in adolescent early blind patients. Interhemispheric functional connectivity was impaired in early blindness, and the duration of blindness negatively correlated with VMHC in the bilateral middle frontal gyri, middle temporal gyri, and inferior temporal gyri. These findings may help researchers understand the pathophysiologic mechanism of early blindness, and these brain areas may be potential target regions for rehabilitation of category-specific visual working memory in early blind patients.

## Figures and Tables

**Table 1:** Demographic data of the early blind subjects.

	Gender	Age (years)	Onset age (months)	Duration of blindness (months)	Clinical description
EB1	M	11.0	0	132	Retinopathy of prematurity; NLP
EB2	M	12.0	0	144	Retinopathy of prematurity; LLP
EB3	F	12.3	0	147	Retinopathy of prematurity; NLP
EB4	M	12.9	0	153	Congenital retinal lesions; eyeball atrophy; NLP
EB5	M	13.3	0	159	Congenital retinal lesions; NLP
EB6	M	14.0	0	168	Retinopathy of prematurity; NLP
EB7	M	14.2	0	170	Retinopathy of prematurity; LLP
EB8	M	14.9	0	177	Retinopathy of prematurity; cataract; NLP
EB9	M	15.0	0	180	Congenital retinal lesions; eyeball atrophy; NLP
EB10	M	15.5	16	169	Fever; LLP
EB11	F	16.2	0	194	Retinopathy of prematurity; NLP
EB12	M	16.6	0	198	Retinopathy of prematurity; eyeball atrophy; NLP
EB13	F	17.0	16	188	Fever; LLP
EB14	F	17.1	0	205	Retinopathy of prematurity; NLP
EB15	F	17.5	0	209	Retinopathy of prematurity; NLP
EB16	M	17.9	0	213	Congenital retinal lesions; NLP

LLP: low light perception; NLP: no light perception.

**Table 2:** Demographic characteristics of the recruited subjects.

	Early blind	Sighted controls	Statistics	*P* value
Gender (male/female)	11/5	11/5	*χ* ^2^ = 0.000	1.00
Age (years)	14.8 ± 2.1	14.9 ± 2.7	*t* = 0.044	0.97
Duration of blindness (months)	175.38 ± 24.54	—	—	—

**Table 3:** Regions showing differences of voxel-mirrored homotopic connectivity between groups.

Brain regions	Brodmann area	Peak (MNI)			Cluster size	Peak *T* value
		*x*	*y*	*z*		
Calcarine cortices	17	±17	−65	13	496	−4.799
Lingual cortices	18	±8	−69	1	456	−4.547
Cuneus	18	±9	−88	18	365	−6.332
Superior occipital gyri	18	±22	−71	24	385	−4.220
Middle occipital gyri	19	±32	−82	17	315	−3.122
Precentral cortices	7	±7	−66	57	517	−4.703

VMHC: voxel-mirrored homotopic connectivity; MNI: Montreal Neurological Institute.

**Table 4:** Regions with significant change in gray matter volume in early blind patients.

Brain regions	Brodmann area	Peak (MNI)			Cluster size	Peak *T* value
		*x*	*y*	*z*		
Left lateral calcarine cortices	17	−16	−73	10	197	−5.077
Left lateral middle occipital gyri	19	−37	−74	6	100	3.456

MNI: Montreal Neurological Institute.

**Table 5:** Regions showing significant correlations between the voxel-mirrored homotopic connectivity and duration of blindness.

Brain regions	Brodmann area	Peak (MNI)			Cluster size	Peak *R* value
		*x*	*y*	*z*		
Middle temporal gyri	21	±62	−35	−10	82	−0.791
Middle frontal gyri	9	±50	−14	43	62	−0.883
Inferior temporal gyri	20	±57	−37	−12	90	−0.844

MNI: Montreal Neurological Institute.
